# Some Examples of the Use of Molecular Markers for Needs of Basic Biology and Modern Society

**DOI:** 10.3390/ani11051473

**Published:** 2021-05-20

**Authors:** Yuri Phedorovich Kartavtsev

**Affiliations:** A.V. Zhirmunsky National Scientific Center of Marine Biology, Far Eastern Branch, Russian Academy of Sciences, 690041 Vladivostok, Russia; yuri.kartavtsev48@hotmail.com

**Keywords:** mtDNA, nDNA, DNA barcoding, genetic introgression, species fate, molecular evolution, reticulation, gene tree, Neo-Darwinism

## Abstract

**Simple Summary:**

The main issues of the report are focused on four items. (1) A combination of nDNA and mtDNA markers best suits the hybrid identification and estimates of genetic introgression between different biological species. (2) The available facts on nDNA and mtDNA diversity seemingly make obvious the introgression presence among many taxa, although, it is evident that introgression may be quite restricted or asymmetric, thus holding at least the “source” taxon (taxa) intact. (3) If we accept that sexually reproducing species in marine and terrestrial realms are introgressed, as it is still evident for many cases, then we should recognize that the biological species concept, in terms of complete lack of gene flow among species, is inadequate due to the fact, that many zoological species are not biological species yet. However, vast modern molecular data proved that with time they definitely become biological species. (4) The recent investigation of fish taxa divergence using central DNA barcoding database shows that most gene trees are, basically, appeared monophyletic and interspecies reticulations are rare.

**Abstract:**

Application of molecular genetic markers appeared to be very fruitful in achieving many goals, including (i) proving the theoretic basements of general biology and (ii) assessment of worldwide biodiversity. Both are provided in the present meta-analysis and a review as the main signal. One of the basic current challenges in modern biology in the face of new demands in the 21st century is the validation of its paradigms such as the synthetic theory of evolution (STE) and biological species concept (BSC). Another of most valuable goals is the biodiversity assessment for a variety of social needs including free web-based information resources about any living being, renovation of museum collections, nature conservation that recognized as a global project, iBOL, as well as resolving global trading problems such as false labeling of species specimens used as food, drug components, entertainment, etc. The main issues of the review are focused on animals and combine four items. (1) A combination of nDNA and mtDNA markers best suits the identification of hybrids and estimation of genetic introgression. (2) The available facts on nDNA and mtDNA diversity seemingly make introgression among many taxa obvious, although it is evident, that introgression may be quite restricted or asymmetric, thus, leaving at least the “source” taxon (taxa) intact. (3) If we consider sexually reproducing species in marine and terrestrial realms introgressed, as it is still evident in many cases, then we should recognize that the BSC, in view of the complete lack of gene flow among species, is inadequate because many zoological species are not biological ones yet. However, vast modern molecular data have proven that sooner or later they definitely become biological species. (4) An investigation into the fish taxa divergence using the BOLD database shows that most gene trees are basically monophyletic and interspecies reticulations are quite rare.

## 1. Introduction

**Figure 1:**
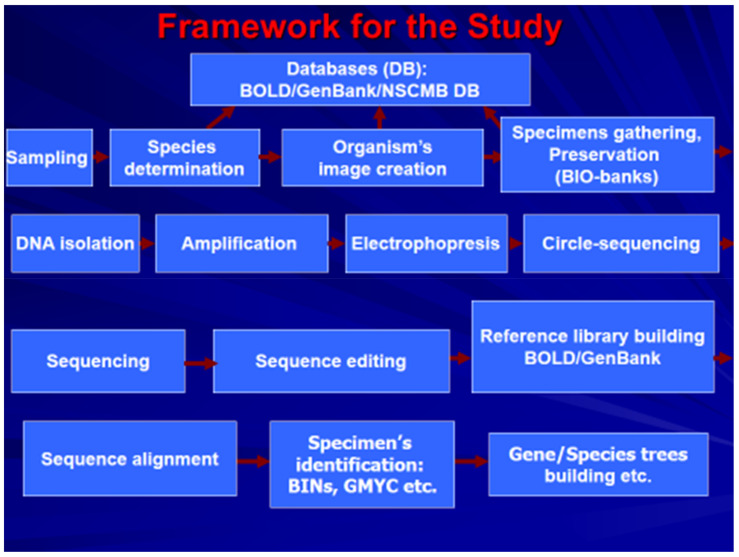
The scheme that illustrates the main steps and activities necessary to develop DNA barcoding and other associated fields of research.

DNA barcoding as a common name of the worldwide initiative, iBOL has been used in biology since 2003 [1,2] (iBOL, the international barcode of life project; www.ibol.org, accessed on 7 August 2020). However, the origin and the application of the approach based on the variability of biological macromolecules, e.g., to systematics and evolutionary biology have a long history [3,4,5,6,7,8]. In general, biological molecular markers (MMs) have found numerous applications to satisfy a variety of needs in modern society. The study and use of MMs have already become a new branch of biomedical science, as evidenced by the establishment of special journals (International Journal of Biological Macromolecules, The Journal of Molecular Diagnostics, Biomarkers, Journal of Current Biomarker Findings, Biomarker Insights, DNA barcodes, etc.). MMs are used in many fields of biology and medicine. Below are the three most important for the subject of this paper; general schedule for DNA barcoding also depicted for convenience (Figure 1).

DNA barcoding. MMs are involved in the global program for a description of biological diversity (biodiversity) on the molecular and bioinformatics platforms. For most invertebrates and vertebrates, a nucleotide sequence (hereafter, the sequence) of the *Co-1* (COI, *cox-1*, etc.) gene, which encodes subunit 1 of cytochrome *c* oxidase of mitochondrial DNA (mtDNA), is used as a standard MM or DNA barcode. For practical needs, the first half of the gene with a length of approximately 650 base pairs (bp) is conventionally used as a barcode. Other MMs or barcodes are more suitable for plants or fungi [9,10]. 

The basis for successful identification of studied eukaryotic species is the low intraspecific variability (low sequence differences between specimens of the same species) but a much higher interspecific divergence of specimens (between specimens of different species). The average intraspecific divergence is approximately 0.5–1.0%, while the interspecies diversity is up to 10%, according to data on animals [11,12,13,14,15,16,17,18] ) that well agrees with pilot findings [1,2,19]. However, that matter is complex and there might be vast heterogeneity of distance scores even among the taxa of a single level, e.g., genera of different organisms [11,12,14] (see discussion in Section 3.3). 

DNA barcoding has many applied use [20]. Recently, one of such appeared due to the globalization and intensification in international trade of food products. The identification of specimens during export and import operations has achieved great importance. Falsified trademarks of seafood such as fish fillets, caviar, etc., can be accurately identified with this kind of MMs, which help customers and retailers avoid significant economical and reputational losses [21,22,23,24,25]. These late matters require special consideration in an assignment or review.

MMs for identification of stocks, lines, and breeds of animals. At this level, *Co-1* and other mtDNA MMs are not quite suitable because of their low variability within a species, as mentioned above (although with some exceptions); MMs of nuclear DNA (nDNA) are usually more conservative in animals and even less applicable at this level. The highest efficiency for the identification of differences between specimens sampled from animal populations, breeds, and lines and for the authentication of single individuals in higher organisms could be obtained by the use of microsatellite DNA loci and single nucleotide substitutions (SNPs). One of MM’s applications is the identification of hybrids and invasive species. 

3. MMs are of greatest importance in medicine (in particular, in diagnostics of such diseases as breast cancer, prostate cancer, colon cancer, etc.) and forensic medicine in particular (exclusion of specimens from suspects in criminalistics). The scope of MM application includes also the monitoring of genetic safety to assess risks of the use of recombinant DNA and genetically modified organisms (cells) in the food and medical industries [10]. A few other applications of MMs, particularly in fishery/aquaculture, were reviewed separately [24,26].

The approximate framework of DNA barcoding and neighboring areas is explained schematically (Figure 1). From this scheme, the importance of the databases (DB) or scientifically defined libraries of properly curated specimens becomes obvious. As is shown in the following sections, this scope influences both societal needs and basic science. The significance of studies described above, besides the obvious areas such as medicine and biodiversity, are particularly important for the validation of biology paradigms, as well as for the iBOL program itself. On August 7, 2020, the iBOL accumulated results of studies on 11,429,832 specimens of living organisms. The number of specimens with barcodes in BOLD (barcode of life database; http://boldsystems.org, accessed on 7 August 2020) is 8,466,913; the number of species identified by barcodes is 314,777 (BOLD; Taxonomic Browser). All these data are accompanied by unified documentation that complies iBOL standards and are freely available via the Internet. The contribution of the Russian Federation and RUS-BOL (http://www.imb.dvo.ru/misc/barcoding/index.htm, accessed on 7 August 2020) to DNA barcoding research in the BOLD on 7 August 2020 (http://www.boldsystems.org/index.php/Public_BINSearch?query=Russia, accessed on 7 August 2020) constitutes 42,174 published records (=records with sequences), forming 7972 barcode clusters (BINs) represented by 263 institutions (laboratories). The records referred to the iBOL Russia node on the above date refer to 27,320 species names, representing 6099 species. By its activity level, the Russian Federation is in the middle of the list of participant states, along with Brazil and France.

For general usage, it should be mentioned the latest DB Midori, a database that verifies the GenBank (www.ncbi.gov, accessed on 13 May 2021) data and able to eliminate incorrect sequences (http://www.reference-midori.info, accessed on 13 May 2021; [27], etc.).

Biodiversity description based on the DNA barcoding approach is successful for the vast majority of taxa, and this fact, along with the ability to delimit species and identify specimens requires explanation and theoretical justification [15,28,29,30,31]. In one of the approaches to understanding the biological basis of this phenomenon, it was proposed to focus primarily on the “pairwise distance” metric, which is equivalent to the *p*-distance or fraction of different nucleotides in a pair of randomly sampled sequences [30] and to evaluate the molecular features of *Co-1* and the whole mtDNA molecule [31]. 

Quite a different approach is considered in this study: the species/specimens delimiting ability is explained mainly by the prevalence of the geographical model of speciation in nature. This model assumes that organisms can accumulate stochastic mutations and unique nucleotide substitutions in DNA chains due to the formation of daughter populations (taxa) under gene flow break between them. With the implementation of this model, specimens of different species are experimentally identified by DNA barcodes, and a correlation of *p*-distances and taxon ranks can be detected by an appropriate analysis [13,14,15,17,28,29,32]. In other words, as shown below with some examples, nucleotide substitutions between specimens of different species could be detected directly in a laboratory as cumulative changes in the sampled DNA/gene nucleotide chains of comparable size.

To address these questions, special consideration of empirical data is required. This consideration must be accompanied by a diversified analysis together with a notion on genetic bases of speciation, as well as with the relevant provisions of the biological species concept (BSC) and, more generally, with Neo-Darwinism, or synthetic theory of evolution (STE). The relevance of this study is also caused by the need to consider the criticism of the BSC/STE paradigms based on the concepts of extensive introgression [33,34,35,36] and reticular evolution [36,37]. These issues have already been considered in part [13,14,15,16,17,28,29,38,39], but still need a more comprehensive quantitative analysis, which is carried out in the present paper.

This study represents an original overview of data and concepts on genetic introgression, reticulation, and a few of mentioned above issues on general genetics and general biology. A new data set that includes up to 12,572 records in MS Excel format is presented for the quantitative meta-analysis of hybrid evidence, genetic introgression, and some records including reticulation data as well (Table S1, Section 2). 

In the review, the analysis of genetic distances, that made before for mostly mtDNA *Co-1* and *Cyt-b* [13,14,15,16,17,28,29,39] is also extended for other MMs like *16S* rRNA and complete mitochondrial genomes (mitogenomes) based on publicly available sequences from GenBank. These latter sequences (*16S* rRNA and mitogenomes) were not used in previous publications on comparative-and-evolutionary analysis of genetic distances along with taxa scaling. The variability of genetic distances in the hierarchy of taxa for the *16S* rRNA gene was first provided in the dedicated literature in Russia and is also considered along with mitogenomes’ data in three specific taxa [40,41,42]. Here these kinds of distance data are combined for a large set of vertebrate mitogenomes as an original impact (details of the analysis are provided in Section 3). 

One of the key objectives of this brief review is to answer the question of whether the available molecular genetic data allow generalizations on the wide presence of genetic introgression between biological species and on the frequent occurrence of reticulation in the studied gene trees, and, if the answer is negative, then, conversely, whether these shreds of evidence are consistent with BSC/STE. STE itself is certainly not a dogma and requires further development. Currently, STE in biology is a general evolutionary concept and, therefore, may be referred to as a theory. However, based on the formal scientific definition, it is unlikely to meet the criteria of a theory. A real theory should include a description in mathematical terms and/or should represent a rigorous model, e.g., a computer model, and have a property to predict events. A consideration of this drawback is available in the relevant literature and some theoretical studies with different levels of generalization appear periodically [12,13,14,15,39,43,44,45,46,47,48,49,50,51].

Data on the possible influence of gene introgression on species evolution, the evolutionary fate of taxa, including reticulations of phylogenetic trees, and the consistency of modern molecular genetic data generally with Neo-Darwinism has been provided in many papers [33,34,35,48,52,53,54,55,56]. 

Concerning the problems of the review, it is important to clarify some phylogenetic terminology in the very beginning. The term “gene tree” was introduced a long time ago [45]. A gene tree is a phylogenetic tree constructed using data for a single gene. This term opposes the concept of the species tree [39] (p. 189), [45,51] (p. 240), which includes a phylogenetic signal for several genes and may incorporate other traits.

A phylogenetic tree, including a gene tree, may have a different topology, including common roots for branches/nodes/clusters (monophyly), or, inversely, have branches with polytomies referred to as para- or polyphyly, indicating reticulation events. Reticulation events or actions are such that cause uncommon descent due to depauperates in Mendelian inheritance, gene or genome duplications, genetic introgression, etc. Some other issues, like sampling errors due to the lack of a signal to resolve a tree topology, are also possible and discussed shortly elsewhere. The topology as well may differ when inferred using different gene (sequences) depending on parameters of lineage diversification. 

There are various controversial issues regarding the BSC/STE. This review focuses mainly on four questions: (1) What methods of identification are most appropriate for the detection of hybrids and genetic introgression, or gene flow? (2) What the facts obtained based on markers of mtDNA and nDNA do indicate? (3) Is there any evidence in the literature for the consistency of molecular variability in phyletic lines or taxa with BSC/STE? (4) How often do reticulations and polytomies of gene trees occur, and what is the main information signal revealed by their topology?

The review analyzes primarily data for animal taxa, but many ideas are applicable also to other taxa, including unicellular organisms.

The general statistical analysis was performed on the basis of MS Excel statistical software and STATISTICA 6.0 [57]. ANOVA/MANOVA, DFA, and other ordinary or multidimensional parametric and nonparametric approaches for statistical testing were widely used. Most details of these tests are explained in publications cited in the review. 

## 2. Estimation of Genetic Introgression: Concept, Terms, and Methods

### 2.1. Notion and Investigation of Hybrids

The content of the section below, as well as in some other parts of the present paper, has been considered recently in a brief overview [16], in the congress proceeding [29], or as a translation of the Russian experimental paper [42]. Currently, the annotation on the subject is provided in the regular issue in this paper, the available base of evidence is widened and I present a quantitative statistical analysis of data (Supplement, Table S1). 

Two concepts are most important for the understanding of the essence of genetic introgression: the notions of hybrid and hybridization. “A hybrid is a genetic mixture, an offspring from a crossing between genetically different organisms”. A specimen with a mixed pedigree, a mestizo, can also be considered a hybrid [51] (p. 151). For testing, a heterozygote of a distant cross for one or more loci could be a hybrid indicator. Hybridization is the process by which hybrids have appeared. Meanwhile, the difference between a simple intrapopulation mating and a crossing for different populations, lines, and broodstocks from one hand, and species from the other should be recognized. In common sense, hybrids are considered to be descendants of more distant crosses. In addition to F_1_, other types of hybrids occur in nature: F_1_ × F_1_ = F_2_, F_1_ × P_1_ = F_b_, etc. 

Hybridization can be artificial or natural. This review is focused primarily on natural hybridization. As noted above, a sharp distinction should be recognized between hybridization and hybrids (hybrid individuals) that occurred afterward artificially or naturally; it is also underlined that hybrids are defined in terms of genotypes that occurred through mating in the natural environment, between normally outbreeding organisms. Agamic, clonal forms, and artificial cases are not considered in this review.

Hybrid individuals may not be exactly intermediate by phenotype between the parental forms and might be closer to one of the parents; in nature, they usually have a decreased fitness compared to that in the parents. The hybrid index, e.g., *I_H_* [53], may be far from 0.5 in this case, and the fitness score may decrease from 1 to a lower level accordingly [12,51] (Ch. 10). (During artificial propagation and broodstocks’ breeding, quite an opposite effect is possible, i.e., heterosis). However, in natural populations, the excess of variability originated from distant crosses often has no positive effect but provides an additional segregation genetic load [12,43,58,59].

From the strict definitions of gene flow [52], it becomes evident that unambiguous detection of hybrids in nature is possible by using nuclear gene markers, which allows identification of the opposite alleles of two parents in the offspring genotype. mtDNA, which is normally inherited in vertebrate animals maternally, can be used with a certain caution. For this reason, the presence in samples of a fragment of mtDNA (or even a complete mitogenome) of Type A specimen in the study of Type B specimens can be explained by a hybridization event that happened in the past. However, a researcher should also exclude other genetic events such as horizontal transfer, recombination, etc. Hybrids obtained only by mtDNA markers should be considered as preliminary identified, since, as noted above, the hybrid genotype identification is certain by the nuclear genes and availability of both parental alleles. 

Evidence for the presence of hybrids may actually reflect the recombination of a region of the mtDNA with nDNA genes. For example, such events were observed in carp fish of the *Gila robusta* complex [55]. Subsequently, the transfer of mtDNA from *Brachymystax lenok* to *Hucho taimen* genome was also described [60]. However, both mentioned examples are only preliminary indices of possible hybridization, because they are entirely based on mtDNA markers. The fact that taxonomically different fishes can interbreed and produce fertile offspring are well known [61]. Researchers [61,62,63,64,65] combined data from more than 4000 studies, including evidence for both artificial and natural hybridization between fish. Some data on this topic were also presented elsewhere [16,29]. In the above papers, among other issues, it was noted that genotypic documentation of hybrids and introgression was not done for a bulk of comparisons. 

It is believed that natural hybridization is more common in fish than in other vertebrates. A similar conclusion may be done for marine invertebrates because the sex chromosome determination system is not well developed for these groups. In a majority of vertebrates, sex determination depends on the determinative gene [66] located on the Y chromosome. However, in fish, sex is determined by several factors and only rarely by the sex chromosomes, which may be completely absent [67]. For example, molecular studies on salmon revealed a weak correspondence of the male’s and female’s phenotypes with sex-specific genetic markers [68]. 

The increased hybridization level in these taxa can be based on several features of the biology of fish and shellfish: external fertilization, weak behavioral isolating mechanisms, unequal numbers of two potential parental species, competition for limited spawning habitats, and, at last, susceptibility to secondary contact of recently diverged forms [53,69,70]. These features can vary significantly depending on local conditions. Natural and human-induced impacts on habitats can be the factors that stimulate fish hybridization [53,64]. Industry-induced changes in ecosystems in North America are also considered as inducing hybridization between the initially allopatric and naturally sympatric pairs of species [71,72,73,74]. For salmon, such examples were reviewed several times [64,75,76]. 

Available sources indicate that hybridization occurred roughly in 25% of plant species and 10% of animal species [33]. In many papers, it is assumed that hybridization per se creates inevitably as a byproduct of genetic introgression. However, this relationship, if it exists, is not simple, as is shown below. Evident cases of genetic introgression are usually observed among young, recently diverged species. For certainty, it is better to focus on the certain notion of species, a kind that better fits BSC, i.e., the biological species by Mayr’s [77] definition, or on similar versions [39] (p. 86), [51] (p. 95), [78]. This choice is important because such species is a reproductively isolated unity and is closest to the population genetic terms considered in this review. Many relevant papers have considered environmental aspects of hybridization [79], impacts of recent historical changes [80], and frequency of natural hybrids in a vast number of taxa: e.g., in birds [81] in comparison with other vertebrates [82,83].

There are at least four methods of hybrid identification: morphological, karyological, biochemical genetics, and molecular genetics [28,29,39,51,53]. MMs are defined as a protein or allozyme, nDNA and mtDNA gene markers most suitable for detection of hybrids and answering questions on the presence of genetic introgression. Detection of hybrids and introgression by the MM analysis of allozyme and nDNA markers is robust if two parental taxa are fixed for different alleles at two or more loci. For instance, for two loci C and D with a pair of alleles each, *C*_1_ vs. *C*_2_ and *D*_2_ vs. *D*_3_, the hybrid individual FH (*C*_1_*C*_2_ *D*_2_*D*_3_) could be well-identified. Two parental taxa are homozygous in this case for different alleles at C and D loci, whereas F_1_ hybrids will be heterozygous for these alleles at two diagnostic loci. However, when hybridization proceeds further than the F_1_ stage, hybrid descendants will give a broad mixture of recombinant genotypes, including genotypes that are identical to the two parental genotypes. In such situations, there is an ultimate necessity for estimating gene flow in precise genetic terms (e.g., Nm, m, Fst, etc.). To date, not so many studies have used such exact population genetic approaches, and the available reviews show examples of some uncertainties [36,37,84]. Even a summary of recent findings gave a lot of empty cells for the key variables, such as Nm and hybrids’ frequency or their type (Table S1, Supplement). A brief look at some numerical outcomes from them will be done in the sections below. 

Empirical data confirm that separate allozymes and nDNA markers are most effective when the parents have different fixed alleles [29,39,51,53] and/or when multi-genomic data are applied [85,86] with estimates of parameters such as Nm, m, Fst, etc. Complex approaches, e.g., the use of mtDNA and nDNA markers can be even more successful as being able to determine, for example, the direction of a parent’s sex in a cross [87]. An integrated approach by MMs and morphometry is also well applicable to assess the genotypic effects, in a particular heterozygote, on the phenotype [88,89,90]. Examples of successful use of MMs combination were reported for turtles of the genus *Mauremys* [85], for cichlids of the genus *Ophthalmotilapia* from Lake Tanganyika [86], for mussels of the *Mytilus* ex. group *edulis* complex with *GLU-5* and other MMs [39,51,89,90,91,92,93,94,95,96,97,98], as well as for many other taxa [87].

To summarize the paragraphs above, it should be stated as follows [29]: 

(1) Hybrid’s identification and detection of genetic introgression are subjects of a major challenge. First, these require accurate genetic analysis with hybrid identification based on many loci and a comparison of descendants of various types (F_1_, F_2_, F_b_, etc.). Subsequently, estimates of allele frequencies, gene flow, and a generalization should be obtained based on these components. 

(2) In this context, BSC is the basic concept for selective testing groups of organisms in genetic terms; intraspecies groups, as well as inbred lines and agamic species (lines of organisms), cannot be considered as representative for understanding the essence of hybridization events and genetic introgression. 

(3) The experimental tools available for analysis in genetics are straightforward and sufficient for hybrid identification and assessment of introgression level.

### 2.2. Genetic Introgression across Species Boundaries

Distinguishing hybrids is often complicated or even impossible if hybridization has been successful and a variety of offspring occurred: F_1_, F_2_, F_b_, etc. When backcrosses or next-generation hybrids are common, the occurrence of recombinant genotypes at a quite high frequency is possible. In such cases, it is difficult to discriminate F_1_ hybrid from a rare parental multiple-locus heterozygote, even when completely diagnostic nuclear loci are used [53]. There are specialized software, e.g., structure (http://pritch.bsd.uchicago.edu/structure.html, accessed on 17 November 2019) [99], DNAsp v5 [100], GENEPOP 3.3 [101,102], MIGRATE-N v 3.0.3 [103,104], SIMCOAL [105], etc., to resolve complicated cases including migration. However, the matter itself and numerical simulations of it are quite sophisticated, and unambiguous population genetic solutions are sometimes impossible to obtain due to the complexity of hybridization events in nature and, especially, in temporal dynamics. The matter considered is even more obscure because there are no precise tools yet to delimit the taxa that have reached and those that have not reached the species rank. In this condition, exact delimitation of species is a very important challenge to evolutionary genetics and evolutionary biology in general [13,14,51,106,107]. The subject is rather complicated, but it is possible to establish an approach that would test a zoological species, which was conventionally identified, with species status for its members identified using MMs, such as the DNA barcode approach with an ID [20] or with a special index, e.g. Barcode Index Number (BIN; BOLD, www.boldsystem.org, accessed on 8 July 2015) or others (see Section 3). 

It seems that among the earliest work using MMs to study the occurrence of hybrids in nature were on fish and shellfish, and particularly on mussel. In the 1980s, the frequency of hybridization among nine sunfish species (genus *Lepomis*) inhabiting two geographic locations in the southeastern USA was assessed using mtDNA markers in combination with allozyme loci [61]. One of the major findings of this research is especially relevant for the current review: no mtDNA or electrophoretic evidence (nDNA base markers) of introgression between the *Lepomis* species was detected; all the hybrids found appeared to be strictly F_1_. Since then, most studies on hybridization phenomena in nature have reported the presence of mostly F_1_ offspring, and also hybrids’ swarms with gene pools merging due to massive introgression, and other cases [34,87].

**Figure 2:**
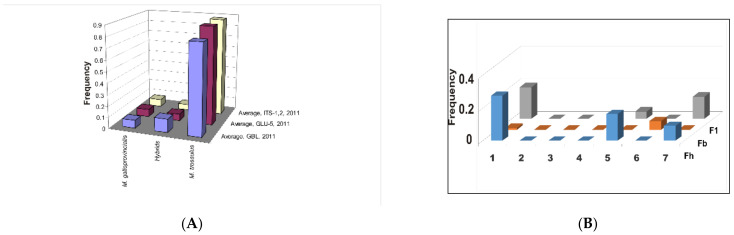
Two histograms of the frequency distribution of the native species *M. trossulus*, hybrids, and the invasive species *M. galloprovincialis* in mussel settlements in the NWSJ (Peter the Great Bay and adjacent waters in the Sea of Japan). The left histogram (**A**) shows a hybrid genotypic group Fh simulated in the Structure software (8 biochemical-genetic loci, GBL, and two nDNA markers, *GLU-5* and *ITS-1,2*). The Y-axis in both figures is the frequencies of genotypes in the total sample from the studied settlements. The right histogram (**B**) shows examples of hybrid occurrence with small or no gene introgression, as evidenced by the decreased score levels for F_b_ offspring. The three rows in figure (**B**) are examples of frequency variation of three types of hybrids (F_h_, F_b_, F_1_) of the two mussel species. On the X-axis are the numbers of samples from mussel settlements in the NWSJ (Modified from [89,98]).

More examples of mtDNA analysis are considered separately below. Prior to this consideration, some data collected by the author’s team for mussels from the Sea of Japan such as the Pacific mussel, *Mytilus trossulus,* and an introduced Atlantic-and-Mediterranean species, *M. galloprovincialis*, are presented. In our first report, combined allozyme-and-morphometric data showed an approximately 5% hybrid occurrence rate in the Sea of Japan waters in Russia, South Korea, and Japan. Hybrid occurrence varied annually within the limit of 1.6 ± 0.9% to 8.9 ± 1.7% [88] (hereafter, values are the mean ± its standard error). The direction of gene flow was determined as *M. trossulus* → *M. edulis* → *M. galloprovincialis* and the species rank of *M. trossulus* was accepted as unambiguous. However, the *M. edulis* and *M. galloprovincialis* taxa were considered to be subspecies/semispecies based on the orthodox BSC [88]. The above conclusion well agrees with the age of taxa, as *M. trossulus* is known to be the most ancient member of the *Mytilus* ex. group *edulis* [88,108]. In the recent reports on these mussels, the genetic variability in the northwestern Sea of Japan (NWSJ) is considered [89,98]. Eight populations were analyzed using eight polymorphic enzyme loci and two nDNA markers (*GLU-5* and *ITS-1,2*). Both enzyme and nDNA markers showed a similar pattern of frequency variation in the two parental species and hybrids. The genotypes of the native Pacific mussel, *M. trossulus,* were predominant, while hybrids were generally rare (Figure 2A). The overall abundance of the invasive species, *M. galloprovincialis,* was relatively low. However, it reached 42 ± 2% in one aggregation, in a sample collected from Possjet Bay off the town of Zarubino, where an international ferry line is operated. The greatest number of hybrids have also been found in this aggregation. 

When searching for the genetic introgression, and assuming the average generation’s length as three years, the Nm rate per generation was estimated approximately at Nm = 5, following Fst rate variation in time. In a different approach, supposing that interspecies gene flow is due to offspring generations such as F_2_, F_3_, and F_b_, rather than from F_1_, the fraction of interspecies migrants, estimated as F_b_ + F_2_, etc., equals 0.9% ± 0.7% (Figure 2B). The obtained evidence suggests a continuous invasion of *M. galloprovincialis* into NWSJ. Judging by the occurrence of hybrids of all types, it is evident that the rate of genetic introgression between two taxa is low, varying over 14 years in the sampled Vostok Bay area (NWSJ) from 0% in 2012 and 2013 [89] up to 8.95 ± 1.68% in 1999 [96]. 

A. Interspecies hybrid frequency sampled for the study of hybrids and introgression in animal taxa. Numerical data are from the sheet Tb-Dt-Plot-Hybr that presents data on hybrids availability the same as in Table S1 (Supplement) but without empty cells. Below the X-axis, numerals denote the ordinal position of taxa listed in Columns 2, 4, and 5 from the sheet Tb-Dt-Plot-Hybr. The numerals from 1 to 21 denote as follows: 1, European butterflies; 2, Flower butterflies; 3, Drosophila; 4, World birds; 5, Grouse of Britain; 6, British ducks; 7, Birds of paradise; 8, American warblers; 9, World tits; 10, Tits; 11, Warblers of Palaearctic; 12, European mammals; 13, Sea stars; 14, Hard clam; 15, Mussels; 16, Eels; 17, Redfish; 18, Sea turtles; 19, Fur seals; 20, Land snails; 21, Breams (scientific names are in column 5 in the sheet Tb-Dt-Plot-Hybr). 

Many theoretical and empirical investigations of hybrid occurrence, hybridization, and genetic introgression, with a variety of other examples available, are summarized elsewhere in the literature [33,37,52,84,109,110,111,112,113,114,115,116,117,118]; some other data can be found in recent author’s overviews ([28,29,42]; see also the Supplement file, Genet-introgr+reticul-evidence-Table1S-etc.xlsx). 

**Figure 3:**
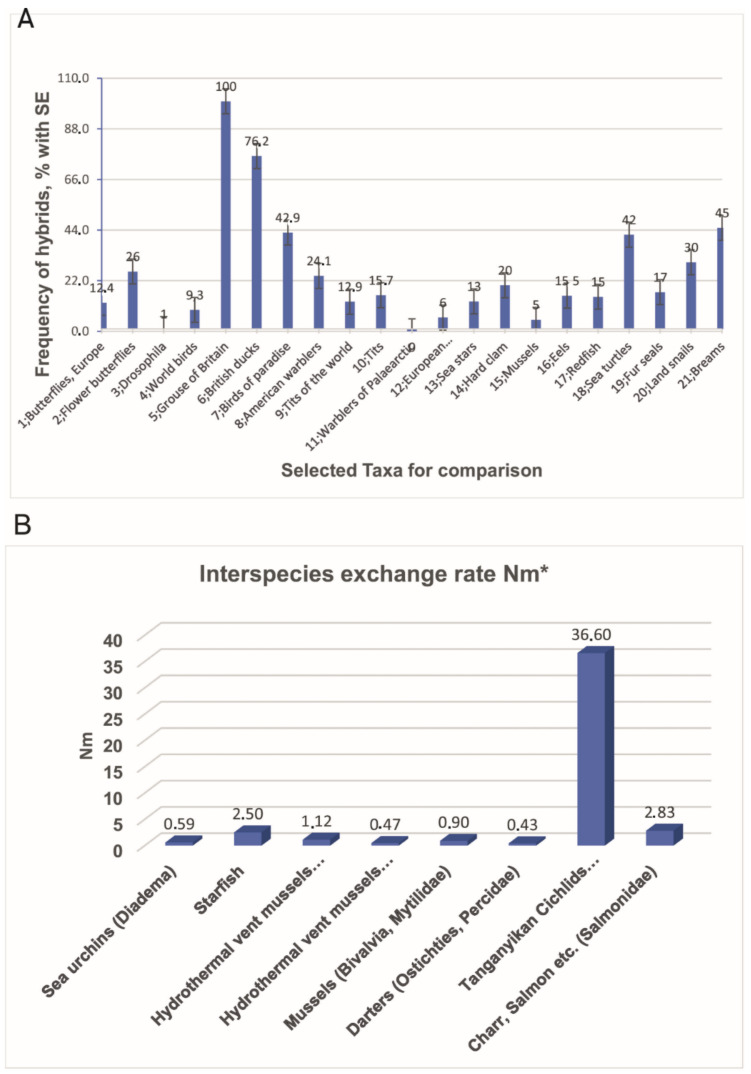
Summary of studies on hybrids and introgression in animal taxa.

Obviously, many aspects of hybridization are obscure and complex, and, thus, some contradictions are possible and may not be resolved soon. For example, in Vertebrates, birds seem to be most prone to hybridization (10–19%, [81,84,119,120]), while amphibians and fish hybridize less frequently but, apparently, with a higher rate than reptiles and mammals [37]. Such an impression may arise due to a biased sampling of different taxa because no statistical evaluation has been made. Unfortunately, no convincing summary statistics are available in cited papers and to the author’s knowledge elsewhere. In the current paper, I tried to fill this gap (Figure 3; see also Supplement, Figures S2 and S3). Particularly, the entire data set includes reanalysis of former reviews’ data and new sources that combine up to 12,572 specimens of animals (Supplement, Table S1, Figure S1 spreadsheets). 

From these data, it is evident that scores of supposed genetic exchanges available as hybrid frequency (F_h_, mostly for F_1_) and Nm vary widely. In the F_h_ rate, the range is from 0 to 100%; in the Nm rate or similar statistics, the range is from 0.4 to 3 (except the score of 36.6 for Tanganyikan Cichlids, as these species hardly fit BSC); the averages of these two variables are as follows: F_h_ =25.7 ± 5.8% and Nm = 5.7 ± 4.7 (Figure 3A,B; Supplement Table S1, and Figure S3A, Figure S3B in sheets Tb-Dt-Plot-Hybr and Tb-Dt-Plot-Nm). Some scores, as noted in comments to Table S1 (sheet Table S1, last column). Many uncertainties, especially for hybrids’ frequency, may arise because represent crosses of an unknown rank, e.g., they may represent intraspecies categories or morphospecies (morphotypes). 

The analysis presented here shows that roughly 2/3 cases identified as genetic introgression are, in fact, F_1_ hybrid occurrence and many are evidence for mtDNA spread across species border but for the nuclear genome an admixture did not notice at the sufficient extent (mtDNA data are commented in the summary Table S1; Supplement: sheet Table S1, last column). After examination of the summary table in mentioned overview by Arnold and Fogarty [36], it became evident that the actual number of cases of genetic introgression is exaggerated, as follows from the discussion in the current paper. 

Let us consider another case, where there is an increase of F_h_ with sample size, and try to discriminate F_h_ presence and the actual genetic introgression in the cases, where the evidence is more or less sufficient (i.e., F_h_ scores are genetically confirmed and fit the conditions for qualitative analysis). A special regression analysis was carried out which provided the conclusion that differs from common expectation: i.e., the greater the species specimens’ number (SSN), the more frequent are hybrids. The relationship that actually obtained is different: SSN negatively related to the hybrid numbers. The variation row for regression analysis has *n* = 16, unfortunately small, due to a large number of empty cells in the original database (Supplement, Table S1 sheet); also, the second variable (SSN) deviates from normality. Despite these weaknesses, it is evident from the score of the Spearman correlation coefficient, which is an appropriate measure for this case, that there is a negative and significant relationship between two variables, F_h_ and SSN: r_s_= −0.7354, *p* < 0.001, *n* = 16. Two bivariate plots with the normal and exponential curves are provided to visualize the relationship (see Supplement, sheet Figure S2. Fh vs SSN; Figure S2A,B). As evident from data in the sheet Figure S2, the Fh vs. SSN correlation is also negative for the Pearson correlation score but is non-significant. Thus, further investigation into this issue is needed. Anyway, obtained data may contradict some of the above-cited views as regards the widespread genetic introgression between species in nature.

According to recent comparative genomics data, the genetic introgression, when thoroughly documented, show examples of mosaic pattern for different parts of the genome (including mitogenome); therefore, it is obvious that many loci remain nearly unchanged even with the certain cases of introgression [86,121,122]. The cases of unidirectional gene exchange [86,123] and the interspecific cytoplasmic gene (mtDNA) flow in the absence of nuclear gene flow that proved long ago are obviously underestimated [124,125]. As already mentioned, Arnold [33] estimated the rate of hybridization among animals as 10%. Taking into account the above presented data, the actual percentage of genetically introgressed animal species might be even smaller, around 5.7%, as illustrated above (with 95% confidence interval from −3.7 to 10.4). Therefore, in support of the goal (1) of the current review, the conclusion is possible that, in spite of consistency or inconsistency to BSC, species as entities in nature are mostly able to maintain their integrity and authenticity, at least in a testable retrospective or provisional perspective. Data presented in Section 3 below should reinforce this idea based on another kind of evidence. 

## 3. The Topology Mode of Gene Trees, Molecular Diversity between Taxa, and Fit of These Data to the BSC/STE and DNA Barcoding Practice

### 3.1. Topology of Gene Trees Inferred from Empirical Data

A number of different modes for building single-gene trees are available, but most have a bifurcative topology (i.e., fork-like splitting of branches in the nodes) with monophyletic or, sometimes, paraphyletic (polyphyletic) branches. Numerical estimations of congruence between the different gene trees do not easy to carry out because their patterns can be different and not comparable. However, phylograms with known branch lengths can be compared, and, when estimated, their congruence is determined as varying and, in many cases, phylogenies match quite well [40,41,126,127]. The bad fit of topologies determined by different genes are also found, but such a pattern usually resulted from the technical-and-informational complications for proper reconstruction. For instance, some discrepancies can occur due to the lack of information capacity of sequences used (small length; e.g., as found for *16S* rRNA in flatfish; [128]) when the number of OTU (operation taxonomic units) being high, as well as due to an inappropriate choice of MM (gene) for the tree reconstruction (too conservative or too variable), and due to other drawbacks in the work with trees [129]. 

In regards to lineage sorting, which objectively can cause different topologies for different genes, a tree built on the whole mitogenome may be more informative then, that on a single gene, as was shown by the results obtained for 100 fish taxa [130], as well as for flatfishes (Pleuronectiformes; [131]) or cyprinid fishes (Cypriniformes, Cyprinidae) [40,132] with the complement of 13 protein genes of the mitogenome. In addition, similar outcomes are reported for many other fish taxa whose representative samples of nDNA genes were used in the analysis [133,134,135,136,137,138]. An approach with numerical simulations and building time-trees for a vast set of candidates’ nDNA loci among Eukaryotes, sampled from 2274 studies representing 50,632 species/specimens of the global time-tree of life, has revealed that genetic diversity basically increases with the rank of taxa [127]. 

There are four main outcomes from the discussed results and from the typical topological signal in gene trees [28,29]: (i) most trees have the evident branch(es) for outgroup; (ii) within taxa of the order rank, major branches/nodes/clusters are represented by families/subfamilies; (iii) lower in the hierarchy, there are well-supported branches that represent different genera of families; and (iv) there are sets of the most close branches comprised of specimens/individuals which are clustered as single-species representatives. A certain fraction of trees contains obscure intragenic and intrafamily clusters that represent usually cases of unresolved topology in some nodes, paraphyly or polyphyly within taxa, with needs for explanation and further examination (usually, later data lead to revisions of taxa in systematics). Let us see how to manage the above data in Section 3.1. 

The latter issue is not easy to resolve. For example, currently, there is no general approach to estimate the number of false neighbors in a cluster from gene trees sampled from studies that are available in the literature. Therefore, no common approach exists to find the degree of reticulation within the trees. Attempts to find a general solution for biodiversity quantification are still made. They are based on several techniques, e.g., on DNA barcoding framework [28,29,30,139], although each of these studies is aimed at different outcomes. Other approaches, such as using Poisson tree processes (PTP) [140], a method similar to the PTP, with generalized mixed yule coalescent (GMYC) theory [141], a comparison of bifurcating patterns in sequence-based species trees [142], and GMYC with the ideology of K/θ-approach [126], were suggested as well. 

A bulk of complications may arise during tree analyses, like those for flatfish *Co-1* and *Cyt-b* gene trees that exhibited paraphyletic intragenic clusters for *Hippoglossoides* and *Pseudopleuronectes* [131] (Figure 1 and Figure 2). However, in the example above, this fact simply reflects morphological misidentifications of some specimens; sometimes, they merely exemplify synonymy of Latin names for a single species, as has been discussed elsewhere [131,143]. The misclassifications mentioned here highlight a problem that is well known in systematics [28,29,139,144] and which usually leads to numerous taxonomic revisions. There is another apparent problem that occurs because of the obscure discrimination of a taxonomic misidentification and the actual false branching in molecular phylogeny, which is caused by genetic reticulation. Beyond the taxa of someone’s expertise, it is usually impossible to resolve that issue even with thorough documentation of such cases. Some other complications are evident, e.g., an mtDNA- vs. nDNA-based tree’s discordance [145], a difference in the rate of substitutions for different genes, a lineage sorting related to Ne variation, and many others created during the genome era in phylogenetics [45,54,146,147].

### 3.2. Congruence between DNA Barcode Data and Conventional Taxonomy Classification

A simple approach was suggested for empirically resolving a topology signal that is based on molecular evidence and classic taxonomy data [28,29]. For this, a concordance is tested between the molecular classifications by DNA barcode data specified as BIN scores and the specimens that zoologically determined by taxonomy experts and gathered in BOLD [28,29]. The BIN score in BOLD is currently defined mostly for *Co-1* sequence data among specified OTUs. BINs are independent of previous taxonomic identifications. Thus, BINs provide means for confirming the match between barcode sequence clusters and species/specimens’ designations by a conventional taxonomy. 

**Figure 4:**
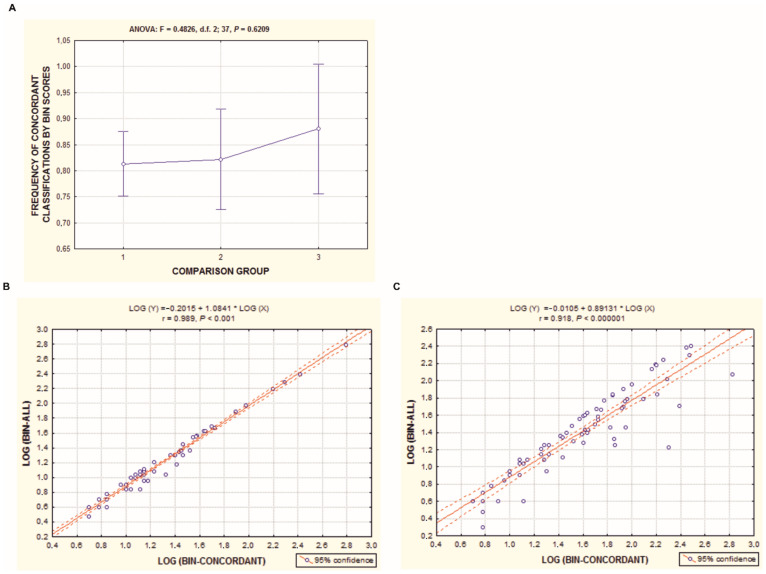
(**A**) ANOVA of BIN score distribution in three fish taxa analyses that were retrieved from BOLD. BIN, barcode index number showing the frequency of concordant classifications (%, Y−axis) of a tested species-specimen by Co-1 mtDNA barcode records and its correspondence to the entire set of records in the BOLD data base that was named in an independent way. The comparison groups (X−axis) are specimens assigned to the certain species (1), genus (2), or family (3). Frequencies of concordant classifications do not differ among the analyzed research projects (see for details [28]); the same also applies to the three comparison groups in the plot (top). The overall average of concordant classifications by BIN estimates is over 82%. (**B**,**C**) Regression analysis of covariation of two variables in the BOLD projects of fish. LOG (BIN-ALL) values (a variable that designates zoologically identified specimens for intraspecies, intragenic, and intrafamily categories as recorded in BOLD, Y−axis) plotted against LOG (BIN-CONCRDANT) scores (all concordant OTU-clusters for *Co−1* mtDNA barcode records or sequence-specimens for the same three categories, X−axis). Variations show a statistically significant positive linear dependence of the two variables for combined projects FERU/TZFPC (**B**) and SCFAA project (**C**). The overall covariation of the two variables for the data set, as estimated by the coefficient of determination on BIN scores, is R^2^ = 98% and R^2^ = 84%, respectively, for two analyses. More details of the analysis are provided elsewhere [28,29].

In the papers cited above [28,29], data were sampled from three fish barcoding projects, TZFPC [148], FERU [149], and SCFAA [150]; species identification and DNA barcoding are based on the expertise of the projects’ authors. These data were selected as close to the authors’ expertise in these fish taxa to minimize complications in interpreting data. Based on BIN scores, it was found that 81.4 ± 2.3% of specimens for species, 84.0 ± 3.9% of specimens for genera, and 88.0 ± 5.8% of specimens for families of these BOLD projects were concordant with the zoological determinations for these fish taxa (Figure 4A). Thus, up to the family level, such molecular marker as *Co-1* that comprises a partial sequence of approximately 600 bp well suits to specimen identifications. In the analysis, no statistically significant differences were observed between the three levels (Figure 4A). More details of the analysis are provided in the cited reports and supplementary data [28]. In the research presented, the LOG-transformed total numbers of BIN scores (LOG-BIN ALL variable, Y-axis that explains specimens’ variation determined by morphology) and the concordant classifications among OTU-clusters for BIN scores (LOG-BIN concordant variable, X-axis) were comparable for the three project’s data (see Figure 4A). In addition, a linear regression and positive correlation for combined data of our own research and other research teams, FERU/TZFPC and SCFAA, was shown [28,29]; see Figure 4B,C). The coefficient of determination (R^2^) for the linear regression function estimated for the two used data arrays is equal to 90% (r_p_ = 0.989, *p* < 0.001, for the least effect). Thus, based on data in Figure 4, a conclusion can be drawn that at the above-considered level (iv) all the sequences/individuals of the same species are determined as single-species clusters, while at level (iii) members of different species are classified into separate genera by their morphology, according to the common practice, with quite high precision. The same is true for the family level (ii), comprised of specimens of different genera. Two kinds of branches at levels (iii) and (iv) sharply differ on any scale of genetic distance [12,13] and, therefore, useful as a tool for DNA barcoding by iBOL and related projects. 

Before proceeding to the following analysis below, where data on the distances calculated directly from sequences or retrieved from trees are used, let us have a look again at the phylogenetic information at the family level (ii), which is valuable for understanding the above-presented correlations on distance vs. topology. An important point for a molecular phylogenetic investigation is family monophyly. 

It is especially valuable for the large one, for instance for such large flatfish families as Pleuronectidae, Soleidae, and Bothidae, for which basic information is currently zoological (e.g., FishBase, etc.); however, relevant data on flounders have also been obtained from molecular phylogenetics [133,134,151]. Monophyly of at least three family-level lineages has been suggested within the Pleuronectoidei, initially based on the results for *12S* and *16S* rDNA [133] and then for *16S* rDNA among several families [134]. Later, a similar paper appeared for *Co-1, Cyt-b*, as well as for the mitogenome data on, mostly, Pleuronectidae [131]. A phylogeny resolving relationship within the Pleuronectidae family is still under development, and recently a wide approach based on several mtDNA and nDNA sequences have been applied [138]. 

Some complications have also been reported: for example, paraphyly in the subfamily Pleuronectinae and paraphyly in the genus *Limanda*, found recently based on larval morphology and molecular markers [131,152]. However, in spite of the data on interspecies hybridization in some flatfish taxa [153], paraphyly even in this fish taxon generally seems attributable rather to the problem of weakness of traits under the morphological determination of individuals (and a habitat change impact in the latter case) than to the origin of the vast hybrid flock in the area of this study in the Baltic. However, such events cannot be ruled out, as it follows, for instance, from discussion in Section 2.2 and data for mussels [97,154]. 

Another large fish lineage, Cypriniformes, has been subjected to even vaster research, including biochemical genetic and molecular phylogenetic approaches [32,40,132,135,136,155,156,157,158,159,160,161]. For these taxa, in spite of numerous occurrences of hybrids, the existence of polyploid forms, and examples of speciation through interspecies hybridization [158,162], most branches obtained in gene trees and mitogenome-based trees indicate that there is no prevalence of genetic reticulation among fishes of the order Cypriniformes. Trees’ nodes there also exhibit mostly a bifurcative type, as is observed in flatfish. In this very diverse taxon, there are certainly problems with molecular systematics that inevitably occur for any big gene tree as compared to small ones. For instance, for the Leuciscinae, big trees have a lower congruence as shown quantitatively by using the Dendroscope software [40]. However, such sort of data is most probably indicating a necessity to increase an information signal from sequences for achieving a better topology resolution for large taxa. 

Beyond the above-mentioned data, there are opposite evidence in support of many genetic reticulations in trees. Such signal comes from trees built for taxa of hybrid zones, as recently found for *Mytilus* ex. group *edulis* (e.g., [154,163,164,165,166], etc.). In addition, complexes of rich tropical/near-tropical fauna give other examples of reticulations [86,167]. There are 17 records of phylogenetic discordance that are summarized in the mentioned review on the introgression impact [36] (Table 1). Being very important, these facts do not alter the general signal on bifurcations and monophyly prevalence in gene trees for a bulk of animal lineages or the ability of MM to delimit fish taxa with a precision of over 80% [28,29,149,167] and other taxa, as clearly evident from the iBOL library. The DNA barcoding evidence relies on the vast empirical BOLD information that validates these conclusions for shallow phylogenies up to the genus level for a vast majority of known eukaryotic taxa and, surprisingly, extends even up to the family level. 

Along with the goals of the review and due to the relevance of population genetics concepts, the BSC is accepted as a framework for key considerations in this paper, despite its applicability to mainly bisexual organisms [47,78,168]. However, for the vast diversity in nature, several other species concepts have also been established, and they are cited here just to provide readers with a list of their authors [47,77,169,170,171,172,173,174,175]. The second important clarification is a keystone to the STE, as summarized by Bush [44], who extended the known Dobzhansky’s [176] concept, the idea on the gene flow break as a crucial factor for the speciation process. Even a further generalization is possible on this issue: “If one could prove that speciation is possible without a gene flow break under a wide genetic exchange, with no gene flow break between lineages, then the BSC/STE must certainly be rejected” [28,29,39,51]. Similarly, it can be stated as follows: if the prevailing speciation modes can create new species without long-term gene pool isolation between parental populations and without reproductive isolation barriers (RIBs), then the BSC/STE paradigms would be disproved? [28,29,39,51]. For the BSC/STE and evolutionary genetics theory, a direct relationship between genetic distance (D) and time (T) is also naturally derived, as clearly defined, for instance, for protein loci due to the accumulation of neutral mutations over time since isolation [45] and in the general case for MMs as a time-dependent coalescent process in lineages [146]. 

From this position, the natural general assumption can be made that MM clusters, including Co-1 barcode, represent species/specimens and must exhibit covariation with ranked taxa that obviously differ in their age from the lowest to the highest. A more focused empirical-and-theoretical consideration of the latter issue is available elsewhere [13,14,28,29,39,51,127]. Certainly, there should be exceptions to the general case where species are single interbreeding populations; such cases include phylogeographic divisions present within species [54] that can have their own complicated evolutionary fate [121,147,177] and those with shared, or overlapping barcode clusters due to a complicated history of species formation and mtDNA introgression ([30] (Figure 2 and Figure 3); see also discussion in Section 2), or even a more complex scenario with introgression-reticulation and genome mosaicism [121]. Actual exceptions from the general assumption that may arise due to the lack of divergence or its small value to be detected by current MM techniques are also expected [178]. For example, if a species originates by the geographic mode (D1) with long-term accumulation of substitutions in isolation, then all the above-explained expectations come true, but when the event of speciation depends on the action of regulatory alterations, chromosomal changes, etc. (speciation modes D3, T2-T4; [46]), then most sampled MMs specimens may become indistinguishable and nearly all DNA barcoding markers will fail.

### 3.3. Molecular Diversity in Taxa of Different Ranks

**Figure 5:**
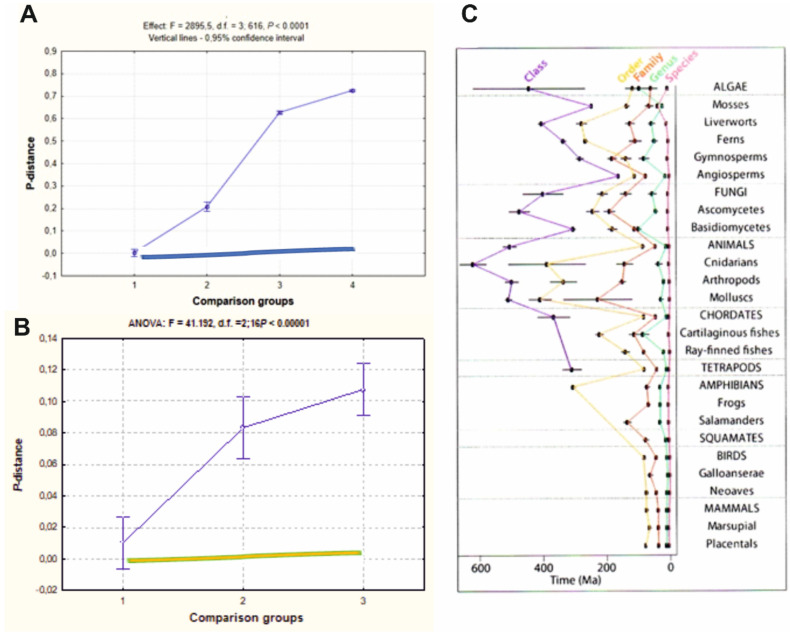
*P*-distance distribution in animal taxa for complete mitogenome in flatfish (**A**), 13 protein gene sequence data of mtDNA in minnow (**B**), and time-dependent variation for nDNA markers in a sample of 50,455 species/specimens of different taxa of Eukaryotes (**C**).

Data representing variation in genetic distances within species and between taxa of different ranks have confirmed that for most organisms, or, more specifically for animals, there is a positive relationship (close to liner for single genes) between two variables: distance score in a comparison group (taxon) and taxa rank [12,13,14,15,39]. This trend is similar for the entire mitogenome (Figure 5; [30]), as well as for the temporal relationships of Linnaean ranks of eukaryotes, showing hierarchical mode for time score and taxa ranks (Figure 5; [127]). Barcode clustering (BIN concordance) and morphology-based taxa ranks (ALL-values concordance) linear relationship (see Figure 4) also should be caused by gradual processes of evolutionary divergence. There are evident exceptions from this rule, showing that gaps between species clusters can be minimal or nearly absent at all [30,40,149]. As a rule, such cases refer to the taxa that have not yet achieved the biological species rank (subspecies, semispecies, or young species), but could also occur due to the presence of species (forms), which are not biological species but, as defined above, originated via genetic transformations, that has not caused (or slightly did) the structural genes per se to be involved routinely in the MMs analysis [12,13,14,28,29,39]. In addition, there are examples of drastic differences in genetic distances among taxa of the same rank for different lineages, like amphibians, birds, and mammals [12,13,14,15,39,54,179]. For this reason, the general DNA barcoding approach and the single scale of genetic distances for the entire biota could not be a universal tool, which fact seems to be overlooked in some summarizing analyses on this topic [30]. There is a way to resolve this complication. In brief, the idea is to use an algorithmic approach based on equations of the set theory after preliminary experimental testing of species/specimens distinctness by means of multiple descriptors of genetic diversity and divergence (with a defined number of estimates of variability and divergence, both for structural and regulatory genes) along with the use of some phenotypic identifiers. Further, it is suggested to carry out special experimental testing of some specimens (individuals) from complicated situations, which can help exclude the cases of conflict: e.g., species vs. modifications [12,13,14,15,39,51].

(A) Variation in the mean values of *p*-distances among four comparison groups for flatfish: (1) *p*-distances inside the species, between individuals of the same species; (2) *p*-distances within the genus, between individuals of different species of the same genus; (3) *p*-distances within the family, between species of different genera of the same family; (4) *p*-distances within the order, between the species of different families of the same order. Color rectangles below main curves in the plots, A and B, show supposed low or absent distance increase along with taxa ranks in a case of no D1 prevalence (after [42] with a modification).

(B) Plot of variation in the arithmetic mean values of *p*-distances among three comparison groups for cyprinids: (1) intraspecies, among individuals of the same species; (2) intragenic, among morphologically distinct species of the same genus; (3) intrasubfamily, among genera of the same subfamily. The effects, i.e., the differences among the mean *p*-distance scores in comparison groups, are exemplified at the top of plots. Bars show a 95% confidence level. Both ANOVA statistical analyses substantiate the intergroup differences (after [40] with a modification).

(C) Temporal relationships of divergence along with Linnaean ranks in Eukaryotes. Taxa grouping is shown on top. The X-axis on the bottom is time-scale. Dots with 95% confidence intervals showing divergence mode among taxa. Vast sample of nDNA markers prove the hierarchical-and-positive dependence of genetic distance and taxa rank (After Hedges et al., 2015 [127]). 

In conclusion, let us have a general look at data on genetic distances for different ranks of taxa. A set of such data have been presented elsewhere for two genes, *Co-1* and *Cyt-b* [12,13,14,15,51] (Chapter 7)); here, new data for complete mitogenome and its 13 protein loci are summarized in Figure 5 [40,42]. All these data allow three main conclusions: 

Conclusion 1. Species delimitation at *Co-1, Cyt-b* and other certain mtDNA gene sequences is highly efficient and unambiguous because of the low intraspecies and high interspecies diversity for these markers [28,29,39,51]. For mitogenome, this statement is valid as well (see Figure 5; A and B, Groups 1 vs. 2). 

Conclusion 2. The positive-and-proportional relationships between distance score and taxonomic rank (Figure 5; [13,14,28,29,39,51,127]) support the idea that speciation in most cases follows the geographic mode (D1 Type, see details in [13,14,39,51] (Chapter 7)) and that phyletic evolution prevails at least in animals. Thus, molecular data empirically prove, on a new level, the basics of the BSC/STE and neo-Darwinian paradigms and their interpretation of speciation and evolution. 

Conclusion 3. As it follows from the obtained and presented data in the discussion, the alternative modes of speciation (D3, T2-T4, etc.) are rare in nature. In the case of alternative hypotheses, i.e., if other speciation modes are equally represented in nature, the relationship between distance and taxa rank should be weakly expressed with small or absent slope (flat); probability of other speciation modes prevalence is certainly disproved by the analysis and other evidence presented in this review (see Figure 5; color rectangles below main curves in the Plots A and B show supposed low or absent distance increase in a case of no D1 prevalence). Such a conclusion does not necessarily mean that other modes are absent or less important. This means rather the well-known fact: Darwinian evolution may prevail with time and provide the current biodiversity of living forms. However, drastic genetic transformations can sometimes produce principal novelties (aramorphosis), although these are rare events in evolution. The latter statement, however, currently have no sufficient empirical or theoretical basement and, therefore, is subjected to debates, which have been continued for many years and has both proponents and opponents. 

Many important issues remained out the consideration because of the limited space of the paper, like the heteroplasmy and mitochondrial pseudogenes presence. Recently serious concerns rose on the heteroplasmy widespread occurrence in many taxa of Eukaryote [180,181] which ignorance may seriously cause conclusions in evolutionary genetics, phylogenetics, and molecular genetics. The impact of pseudogenes’ presence in a genome may be also underestimated [182] and needs a special investigation.

## Data Availability

Not applicable.

## Supplementary Materials

The following are available online at https://www.mdpi.com/article/10.3390/ani11051473/s1.

## Abbreviations

F_1_: F_2_, F_b_	denoted hybrids of first, second generations, and backcrosses, correspondingly
MM	biological molecular marker (markers, MMs)
iBOL	the international barcode of life project; www.ibol.org (accessed on 7 August 2020)
BOLD	barcode of life database; http://boldsystems.org (accessed on 8 July 2015)
mtDNA	mitochondrial DNA
nDNA	nuclear DNA
mitochondrial genome	mitogenome
*Co-1* (COI, *cox-1*, etc.)	gene, which encodes subunit 1 of cytochrome *c* oxidase mtDNA
*Cyt-b* (cytb, etc.)	gene, which encodes cytochrome b mtDNA
Bp	base pairs
SNPs	single nucleotide substitutions
