# Supplementary figures and images for: Some Examples of the Use of Molecular Markers for Needs of Basic Biology and Modern Society
Source: Animals (Basel). 2021 May 20;11(5):1473. doi: 10.3390/ani11051473 (PMC8160991; doi:10.3390/ani11051473)

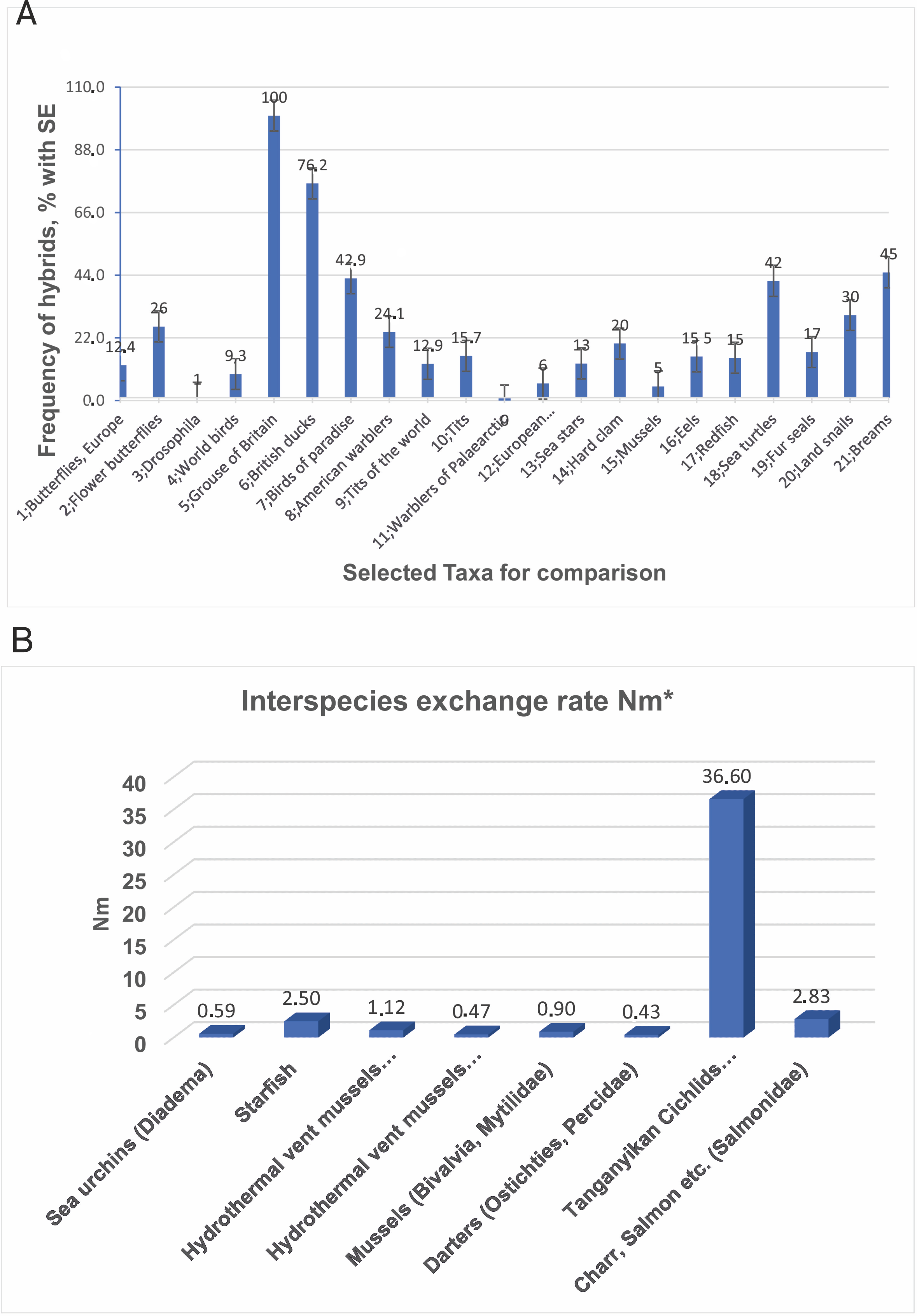

Supplement: Supplementary file 1 [file animals-11-01473-s001.zip › Figures-Kartavtsev/Fig. 3fin.png]

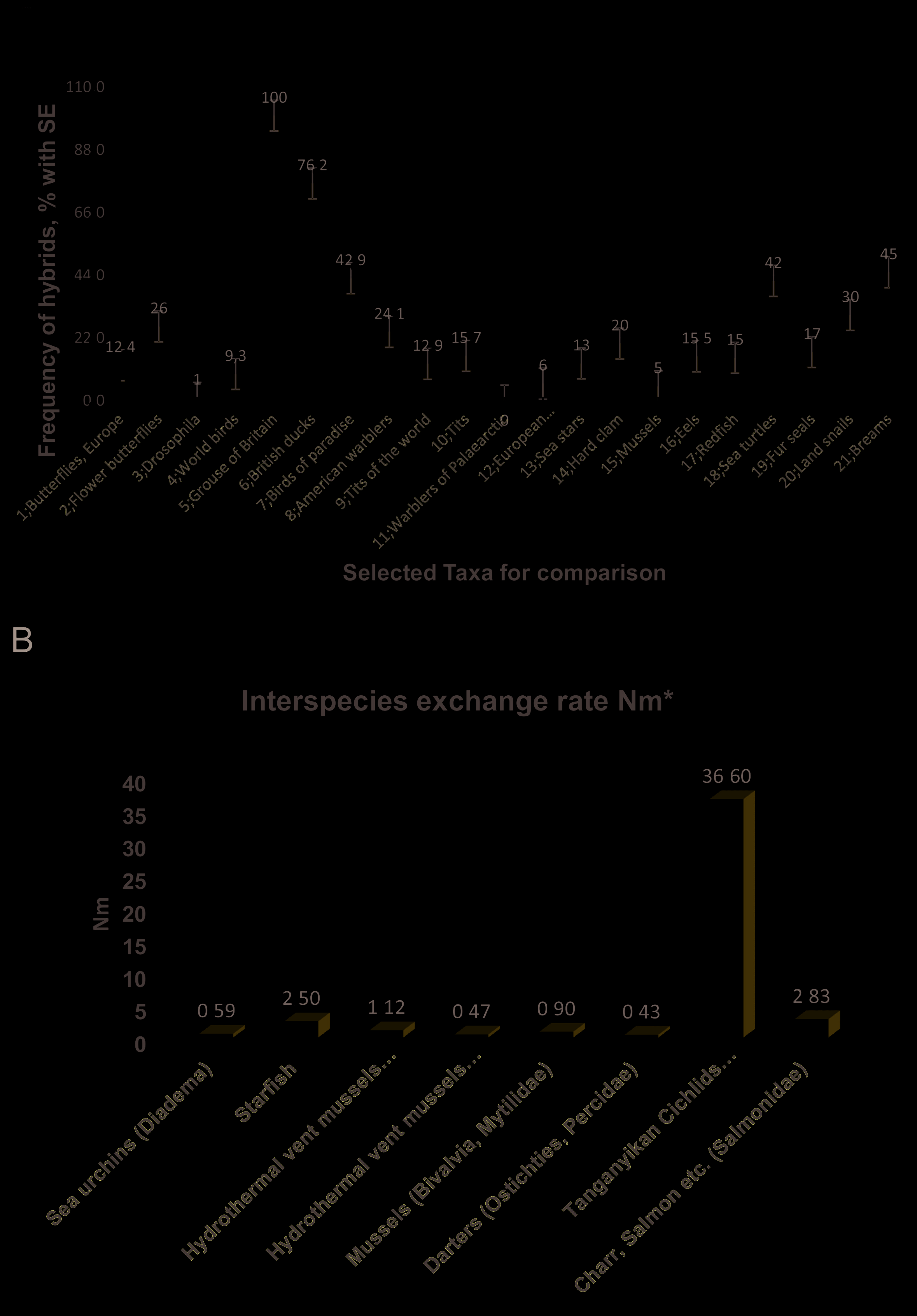

Supplement: Supplementary file 1 [file animals-11-01473-s001.zip › Figures-Kartavtsev/Fig. 3fin.tif]

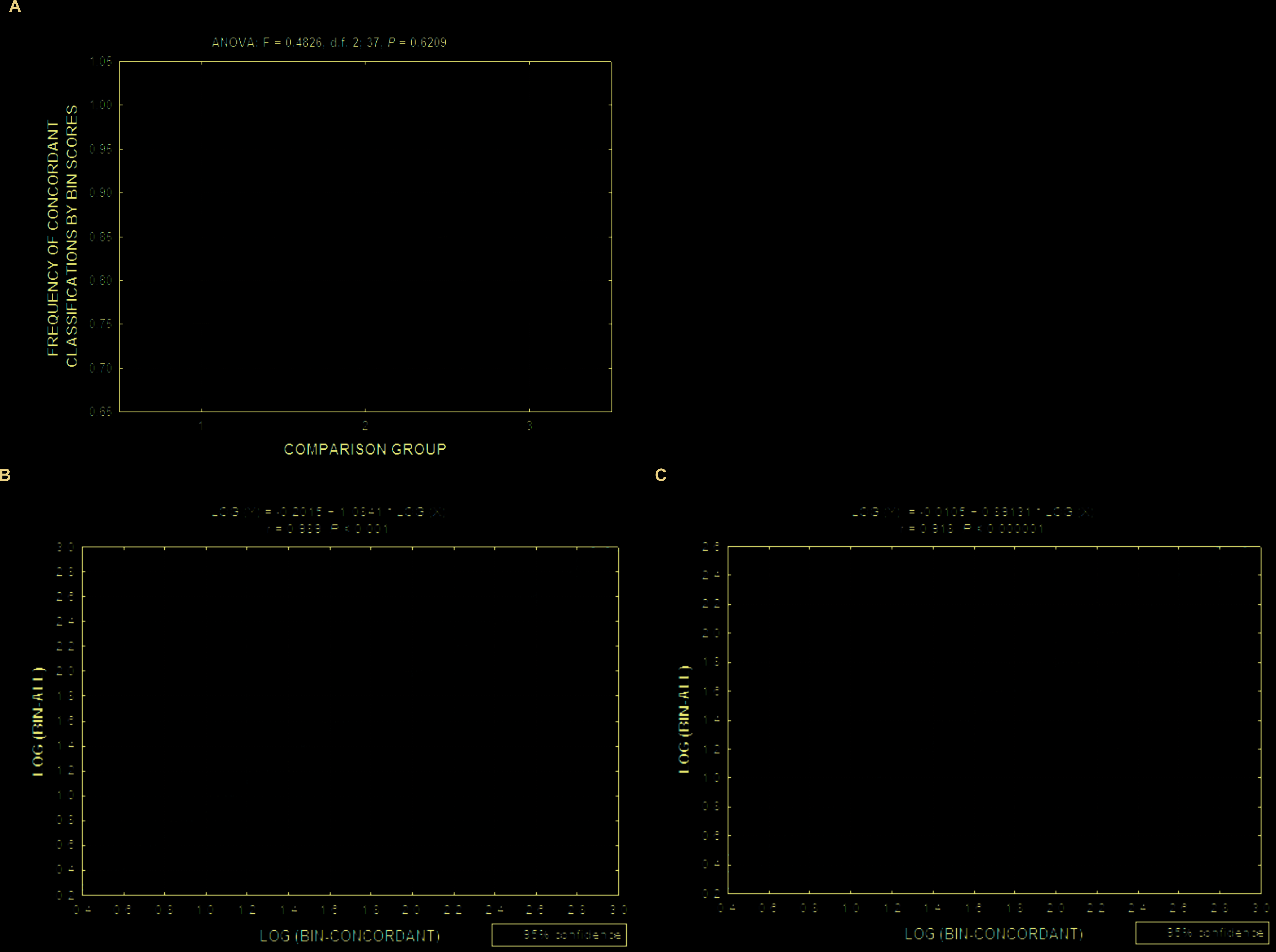

Supplement: Supplementary file 1 [file animals-11-01473-s001.zip › Figures-Kartavtsev/Fig. 4f.tif]

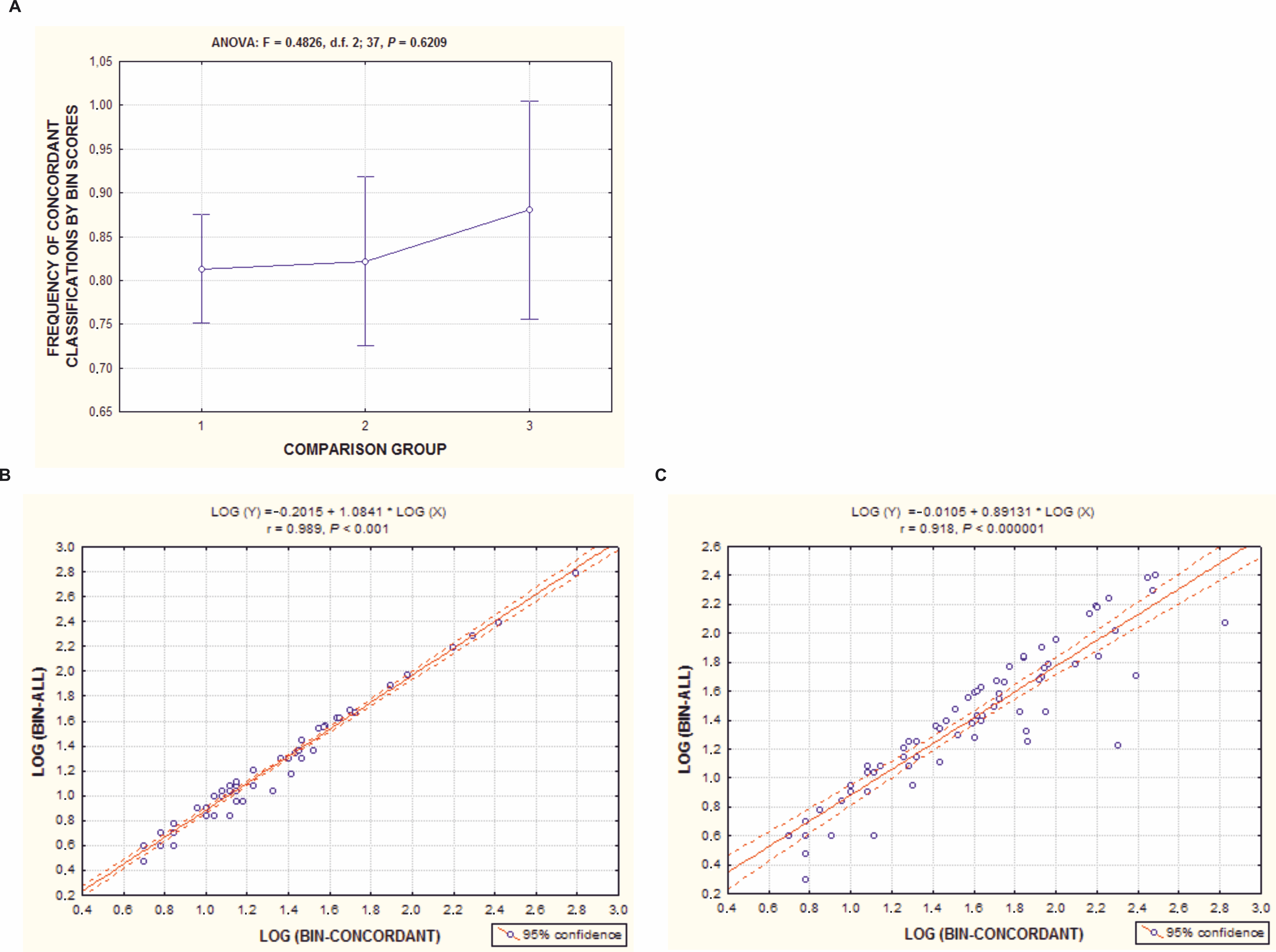

Supplement: Supplementary file 1 [file animals-11-01473-s001.zip › Figures-Kartavtsev/Fig. 4fin.png]

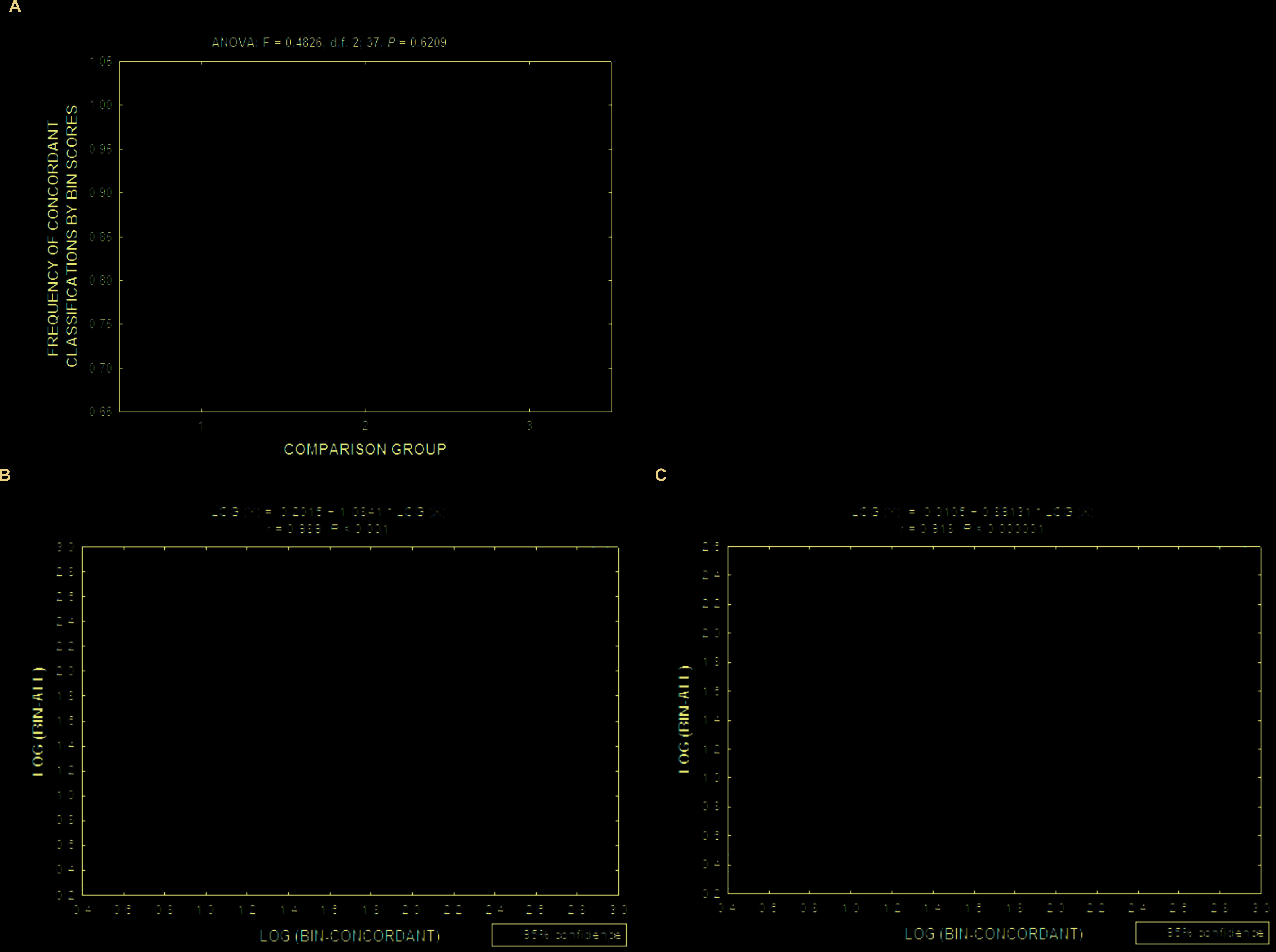

Supplement: Supplementary file 1 [file animals-11-01473-s001.zip › Figures-Kartavtsev/Fig. 4fin.tif]

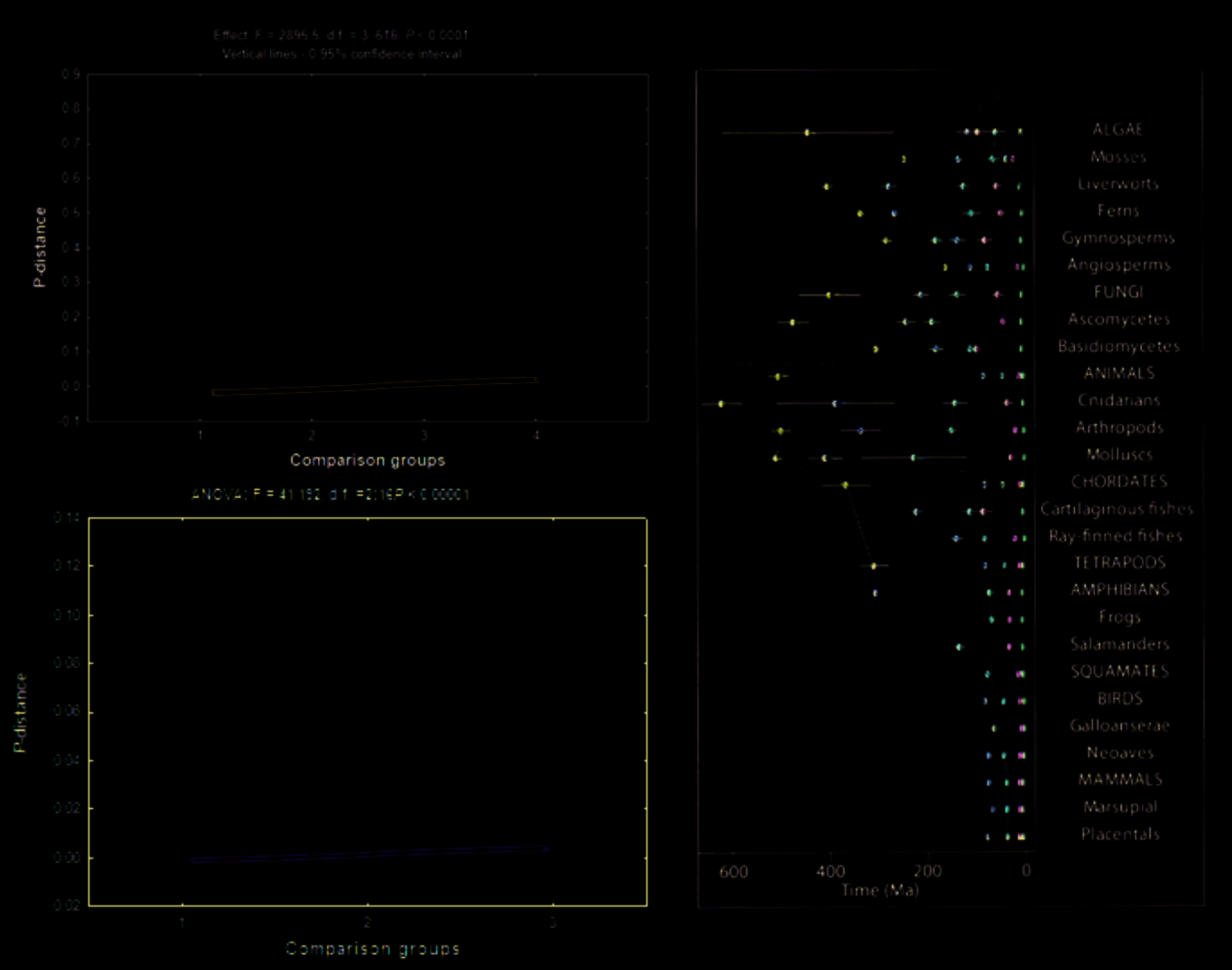

Supplement: Supplementary file 1 [file animals-11-01473-s001.zip › Figures-Kartavtsev/Fig. 5f.tif]

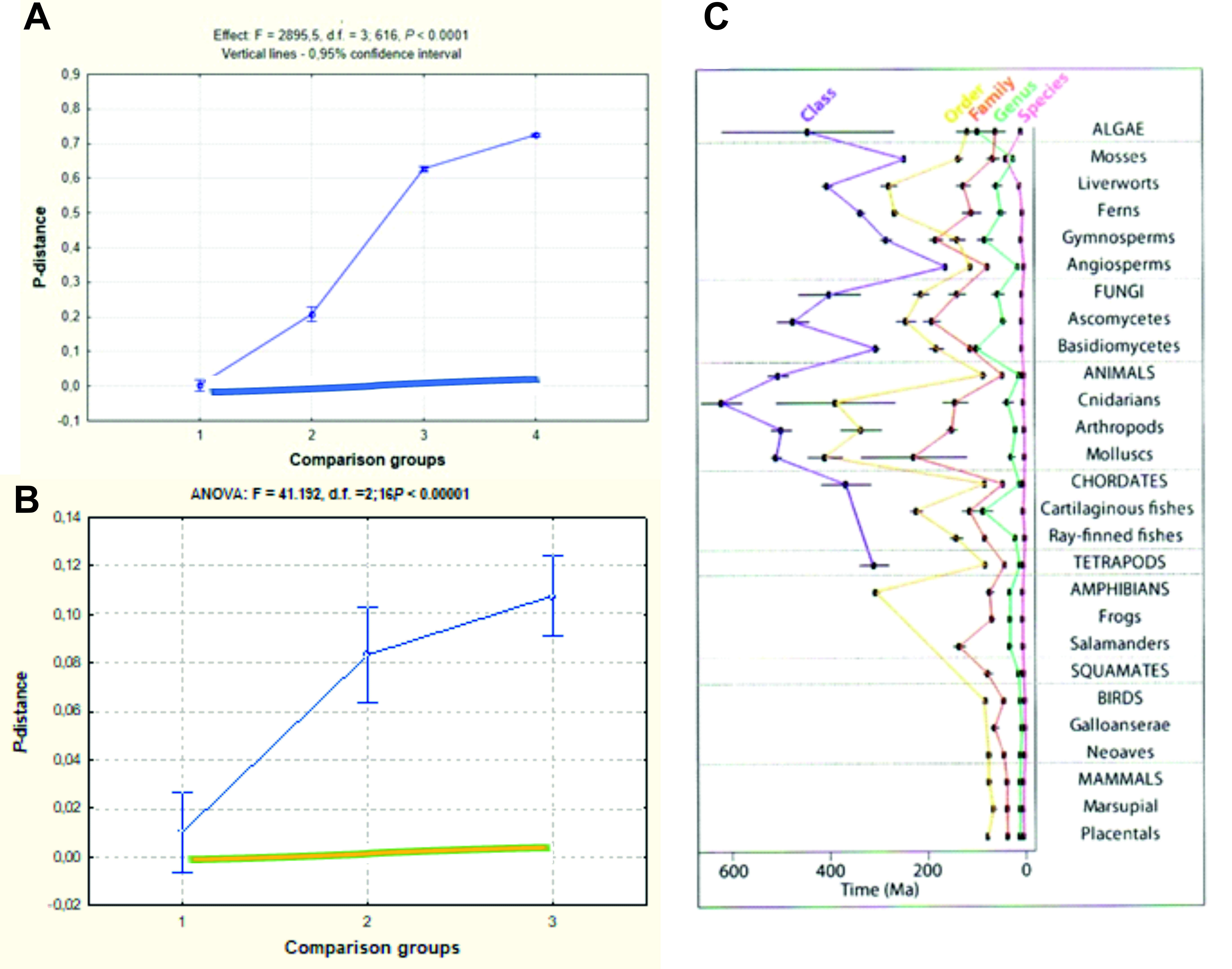

Supplement: Supplementary file 1 [file animals-11-01473-s001.zip › Figures-Kartavtsev/Fig. 5fin.jpg]

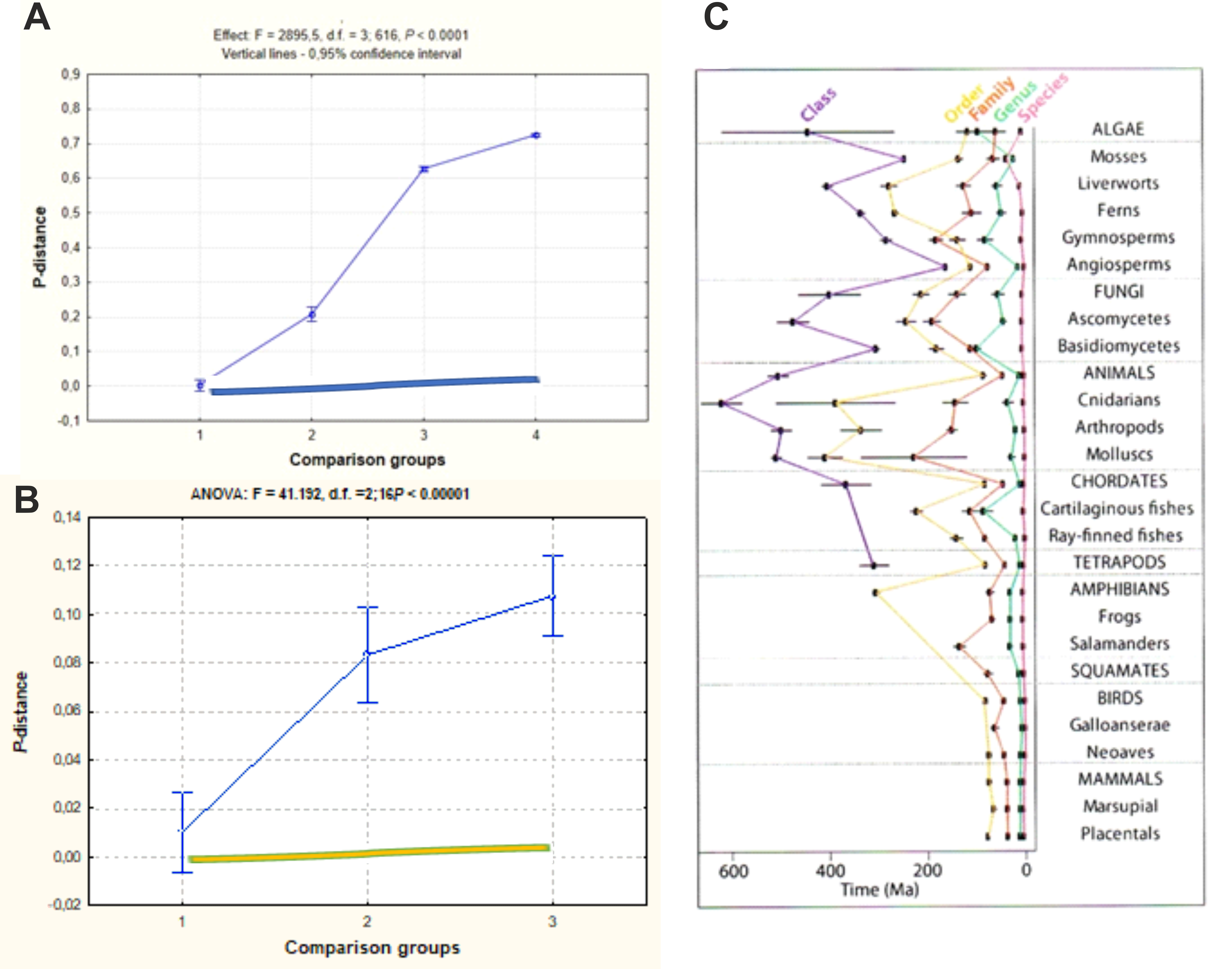

Supplement: Supplementary file 1 [file animals-11-01473-s001.zip › Figures-Kartavtsev/Fig. 5fin.png]

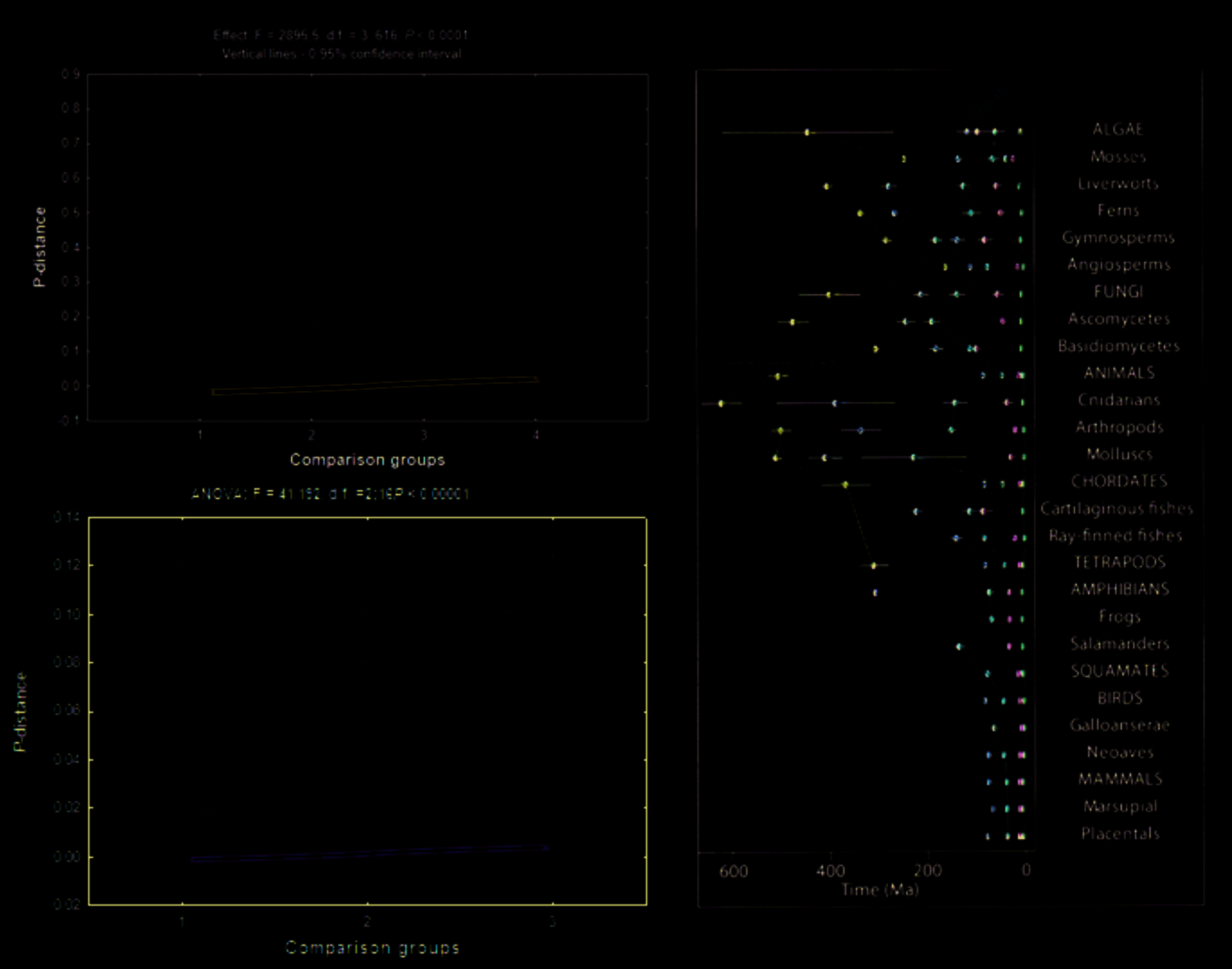

Supplement: Supplementary file 1 [file animals-11-01473-s001.zip › Figures-Kartavtsev/Fig. 5fin.tif]
